# Identification and validation of an immune-related lncRNAs signature to predict the overall survival of ovarian cancer

**DOI:** 10.3389/fonc.2022.999654

**Published:** 2022-10-12

**Authors:** He Li, Zhao-Yi Liu, Yong-Chang Chen, Xiao-Ye Zhang, Nayiyuan Wu, Jing Wang

**Affiliations:** ^1^ The Animal Laboratory Center, Hunan Cancer Hospital and The Affiliated Cancer Hospital of Xiangya School of Medicine, Central South University, Changsha, China; ^2^ The Central Laboratory, Hunan Cancer Hospital and The Affiliated Cancer Hospital of Xiangya School of Medicine, Central South University, Changsha, China; ^3^ Department of Gynecologic Cancer, Hunan Cancer Hospital and The Affiliated Cancer Hospital of Xiangya School of Medicine, Central South University, Changsha, China

**Keywords:** ovarian cancer, immune-related lncRNA, prognostic signature, chemotherapy, risk score

## Abstract

Ovarian cancer (OC) is the most lethal gynecological cancer in women. Studies had reported that immune-related lncRNAs signatures were valuable in predicting the survival and prognosis of patients with various cancers. In our study, the prognostic value of immune-related lncRNAs was investigated in OC patients from TCGA-RNA-seq cohort (n=378) and HG-U133_Plus_2 cohort (n=590), respectively. Pearson correlation analysis was implemented to screen the immune-related lncRNA and then univariate Cox regression analysis was performed to explore their prognostic value in OC patients. Five prognostic immune-related lncRNAs were identified as prognostic lncRNAs. Besides, they were inputted into a LASSO Cox regression to establish and validate an immune-related lncRNA prognostic signature in TCGA-RNA-Seq cohort and HG-U133_Plus_2 cohort, respectively. Based on the best cut-off value of risk score, patients were divided into high- and low-risk groups. Survival analysis suggested that patients in the high-risk group had a worse overall survival (OS) than those in the low-risk group in both cohorts. The association between clinicopathological feathers and risk score was then evaluated by using stratification analysis. Moreover, we constructed a nomogram based on risk score, age and stage, which had a strong ability to forecast the OS of the OC patients. The influence of risk score on immune infiltration and immunotherapy response were assessed and the results suggested that patients with high-risk score might recruit multiple immune cells and stromal cells, leading to facilitating immune surveillance evasive. Ultimately, we demonstrated that the risk model was associated with chemotherapy response of multiple antitumor drugs, especially for paclitaxel, metformin and veliparib, which are commonly used in treating OC patients. In conclusion, we constructed a novel immune-related lncRNA signature, which had a potential prognostic value for OC patients and might facilitate personalized counselling for immunotherapy and chemotherapy.

## Introduction

Ovarian cancer (OC) is the most lethal gynecological cancer among women in worldwide, with 313,959 estimated new cases and 207,252 new deaths in 2020 (1). Due to the ambiguity of early symptoms and the lack of reliable screening strategies, more than 60% OC patients are diagnosed with later-stage. Complete cytoreductive surgery followed by platinum-based chemotherapy is known as the standard first-line treatment protocol for OC patients. However, a high proportion patient will relapse within 2 years of diagnosis (2). Therefore, there is an urgent need to identify prognostic biomarkers to predict the outcome of OC patients.

It is being increasingly recognized that immune system plays vital roles during cancer initiation and progression (3). Moreover, it is suggested that tumor progression and invasion is dependent on intratumoural adaptive immunity and the immunological type, density, and location of immune cells within the tumor samples are superior to TNM staging in predicting the natural history of primary cancers (4, 5). It has been reported that patients whose tumors with more tumor-infiltrating lymphocytes (TILs) predicted longer survival in OC. Besides, recruitment of T-regulatory (Treg) cells in OC can foster immune privilege and predict reduced OS (6, 7). All the evidence convincingly indicated that OC was an immunogenic tumor (8). Therefore, the immune-related prognostic signature might be a potential tool to predict outcome of OC patients.

Long non-coding RNAs (lncRNAs) are a group of RNA molecules whose transcripts are greater than 200nt but not translated into proteins. They participate in various biological progress, such as epigenetic regulation, genetic imprinting, chromatin organization and protein modification (9, 10). Moreover, they participate in immune response including antigen presentation, antigen release, immune cell differentiation and T cells infiltration (11, 12). Lnc-EGFR stimulates Treg cells differentiation and promotes immune invasion in hepatocellular carcinoma (13). Lnc-DC, which is a specific marker of dendritic cells (DCs), promotes the ability of DCs to active T cells (14). LincR-Ccr2-5’AS increases the migration ability of Treg cells (15).

In OC, a new lncRNA small nucleolar RNA host gene 12 (SNHG12) was proved to promote immune escape of OC cells through their crosstalk with M2 macrophages (16). Moreover, lncRNA HOTTIP was suggested to promote the secretion of IL-6 and up-regulate the expression of PD-L1 in neutrophils, leading to the inhibition of T cells activity and acceleration immune escape of OC cells (17). Recently, it was demonstrated that lncRNA XIST could affect the cell proliferation and migration *via* mediating macrophage polarization in both breast cancer and OC (18). In addition, FOXP4-AS1 and MEG8 were revealed to be associated with immune infiltration in OC (19, 20). All these evidences indicated that immune-related lncRNAs played important roles in OC.

Recently, multiple immune-related lncRNA signatures have been identified to predict the OS in various cancers, including breast cancer (21–24), hepatocellular cancer (25), lung cancer (26), cervical cancer (22, 27), colon cancer (28), glioma (29–31), and bladder cancer (32, 33). However, the immune-related prognostic lncRNA signature for predicting the prognosis of OC patients has not been developed. In our study, we aimed to explore the prognostic value of the immune-related lncRNAs in OC and validate an immune-related prognostic lncRNA signature for patients with OC.

## Materials and methods

### Data acquisition and preprocessing

For TCGA-RNA-Seq training set, mRNA gene expression profiles and corresponding clinical information were downloaded from the TCGA data source (https://xena.ucsc.edu). To increase the statistical power and overcome the systematic errors caused by small sample size, we combined the datasets (GSE26193, GSE30161, GSE63885, GSE9891, GSE18520 and GSE19829) with the HG-U133_Plus_2 platform as the HG-U133_Plus_2 validation set (34–39). All clinical information and microarray data were captured from GEO repository (https://www.ncbi.nlm.nih.gov/geo/). Ultimately, we obtained a TCGA-RNA-seq training cohort with 378 patients and a HG-U133_Plus_2 validation cohort with 590 patients.

### Identification of immune-related lncRNAs

The lncRNA annotation file was acquired from the GENCODE website for annotation of the lncRNAs. Consequently, 14826 lncRNAs and 2448 lncRNAs were identified from TCGA-RNA-Seq cohort and HG-U133_Plus_2 cohort, respectively (40). The immune-related genes were obtained from the ImmPort database (http://www.immport.org) (41). Pearson correlation analysis was utilized to screen immune-related lncRNAs. Those lncRNAs with r>0.3 and *p<0.001* were considered as immune-related lncRNAs (25). To assess the prognostic value of immune-related lncRNAs, we further conducted univariate Cox regression analysis by using the “survival” package, and the hazard ratios (HR) with 95% confidence intervals (CIs) were examined. *p < 0.05* was considered that immune-related lncRNAs were significantly correlated with OS) and served as prognostic immune-related lncRNAs.

### OS analysis

OS was defined as the time from randomization to death from any cause. The survival curves were calculated and illustrated by the KM plot with the long-rank test.

### Construction of immune-related prognostic lncRNA signature

Based on the prognostic immune-related lncRNAs, a risk signature was constructed by using the “glmnet” package (42). Through 1000 cross-validation, a panel of genes and their LASSO coefficients were obtained. The risk scores for the signature were calculated using the following formula: Risk score=β1X1+β2X2+⋯+βnXn (β, LASSO coefficient; X, the expression of each prognostic immune-related lncRNA in each sample). Based on the best cut-off value of risk score, patients were divided into high-risk and low-risk groups. Kaplan–Meier method with the long-rank test were performed to reveal the OS of the high-risk and low-risk groups by using the “survival” package. Besides, time-dependent relative operating characteristic (ROC curve) and area under the curve (AUC) were applied to assess the prediction ability of the signature. All the time-dependent ROC curves were calculated and drew by “SurvivalROC” and “ggplot2” package, respectively.

### Decision tree and prognostic nomogram construction

Decision tree and nomogram model were applied to define significant clinical predictors. Firstly, univariate and multivariate COX regression were performed to select important explanatory variables. Based on the multivariate cox regression results, stage, age and risk score were identified as predictor variables. After then, the “rpart” Package (https://cran.r-project.org/web/packages/tree/index.html) was used to construct decision tree and split patients as different from each other as possible. It was implemented to decide which of these variables to split and the splitting value in each step of the tree’s construction (43). Moreover, a nomogram model, which is an individualized risk prediction model to predict the 1, 3, 5-year survival probability, was constructed using the “RMS” package. The calibration curves were used to assess the concordance of the observed and predicted rates of 1, 3, 5-year OS (44).

### Estimation of tumor-infiltration, immunotherapy and chemotherapy response

Firstly, the ESTIMATE algorithm (https://bioinformatics.mdanderson.org/public-software/estimate/), which can be applied for assessment of the presence of stromal cells and the infiltration of immune cells in tumor samples using gene expression data, was used to calculate the Estimate score, Immune score, Purity score and stromal score (45). Briefly, we defined ssGSEA based on the signatures related to stromal tissue and immune cell infiltration as Stromal score and Immune score, respectively, and combined the stromal and immune scores as the ESTIMATE score. Purity score was calculated as followed: Purity score= cos (0.6049872018 + 0.0001467884*ESTIMATE score). The correlation of risk score and Estimate score, Immune score, Purity score and stromal score were analyzed by using Pearson correlation analysis. The infiltration of 22 subtypes of tumor-infiltrating immune cells (TIICs) was acquired from CIBERSORT algorithm (http://cibersort.stanford.edu/) (46). Tumor Immune Disfunction and Exclusion (TIDE) algorithm (http://tide.dfci.harvard.edu/), which is a method to accurately predict the outcome of patients treating with immune checkpoint blockade (ICB), were employed to evaluate the immunotherapy response (47, 48). The chemotherapy response was evaluated by using the Genomics of Drug Sensitivity in Cancer database (GDSC, https://www.cancerrxgene.org). The half-maximal inhibitory concentration (IC50) of all drugs commonly used to treat tumors were calculated and represented the drug response. The R package ‘pRRopheticRredic’ was used with 10fold cross-validation and other parameters by default (49).

### Exploration of immune-related lncRNA function

To further explore the function of the five immune-related lncRNA, we firstly assessed the association between the five immune-related lncRNA and immune-related mRNA by using Pearson correlation analysis. Then, the results were converted visually and the co-expression network was identified with Cytoscape software (50). Based on gene expression or the risk score, patients were divided into two groups. GSEA assay was utilized to explore whether a series of priori defined pathways were enriched in the gene bank derived from DEGs between the two groups (51, 52). FDR<0.05 was identified as enriched. Moreover, the absolute immune scores from gene expression datasets were obtained by LM22 (22 immune cell types) gene signatures of CIBERSORT algorithm (46). The molecular immune cell subtypes related to the five lncRNAs expression were captured by using Spearman correlation analysis (53). Only *p<0.05* was considered significant.

### Cell culture, RNA extraction and real-time quantitative PCR

OC cell lines, SKOV3, A2780, OVCAR8 and OVCAR3, were obtained from Institute of clinical pharmacology, Central South University. All the cell lines were cultured in RPMI-1640 medium with 10% FBS. All cell lines were cultivated at 37°C and 5% CO_2_. Total RNAs were extracted from OC cell lines by using Trizol reagent (Takara). After extraction, total RNAs were reverse-transcribed into cDNA using PrimerScript™ RT reagent Kit (RR047A, Takara). Real-time quantitative PCR was performed using the SYBER Premix Ex Taq kit (RR420a, Takara) in Roche-LightCycler 480 system (Roche,USA). Finally, the relative expression of lncRNAs were calculated based in the internal reference GAPDH. The primers of lncRNAs and GAPDH are listed in **supplementary Table 1**.

### Lentivirus infection

The packaged lentivirus vectors of UBXN10-AS1 overexpression (LV-BUXN10-AS1) and empty lentivirus vectors (LV-NC) were purchased from GenePharm (Shanghai, China). For UBXN10-AS1 overexpression, the LV-UBXN10-AS1 or LV-NC were introduced in SKOV3 and A2780 cells at an MOI OF 50-100. After 72h post-infection, the infection efficiency was measured by using RT-qPCR.

### Cell proliferation assays

Cell proliferation was assessed by using CCK8 kit (MCE, China). Briefly, cells (1-2 *10^4^ cells/well) infected with LV-UBXN10-AS1 or LV-NC were seeded into 96-well plates and cultured in a CO_2_ incubator for 24,36, 48,72 and 96h. Subsequently, 10μl of CCK8 reagent was added into the wells and the plate was incubated for 1h. Finally, the OD value was measured at 450nm using the microplate reader (54).

### Cell migration assay

To detect the cell migration, wounding healing assay was performed. Firstly, cell infected with LV-UBXN10-AS1 or LV-NC were seeded in 24-well plates. A 200ul pipette tip was used to scratch the cell layer, when cells reached 70-80% confluence. Cells were grown for an additional 48h. Microscope images were captured at 0h and 48h (55).

### Annexin V/PI apoptosis assay

Cells were plated in 6-well plate with 1*10^5^ cells/well. After 12 hours, cells were infected with lentivirus vectors. After 48 hours incubation, cells were harvested, washed with PBS and incubated with Annexin V and PI, using the Annexin V-APC apoptosis detection kit (KGA1022, KeyGen, China). The flow cytometry analyses were performed with CytoFLEX instrument (Beckman Coulter, USA).

### Statistical analysis

The two‐tailed Students’ t-test was utilized to analyze the significant differences between groups, whereas quantitative differences among groups were analyzed by using the one‐way ANOVA. Kaplan–Meier curves and log-rank test were implemented to calculate the OS rate. All statistical analyses were performed using R software (version 3.6.2). * means *p<0.05*, ** means *p<0.01*, ***means *p<0.001*. *p<0.05* was considered statistically significant.

## Results

### Identification of immune-related prognostic lncRNAs in OC patients

**Figure 1:**
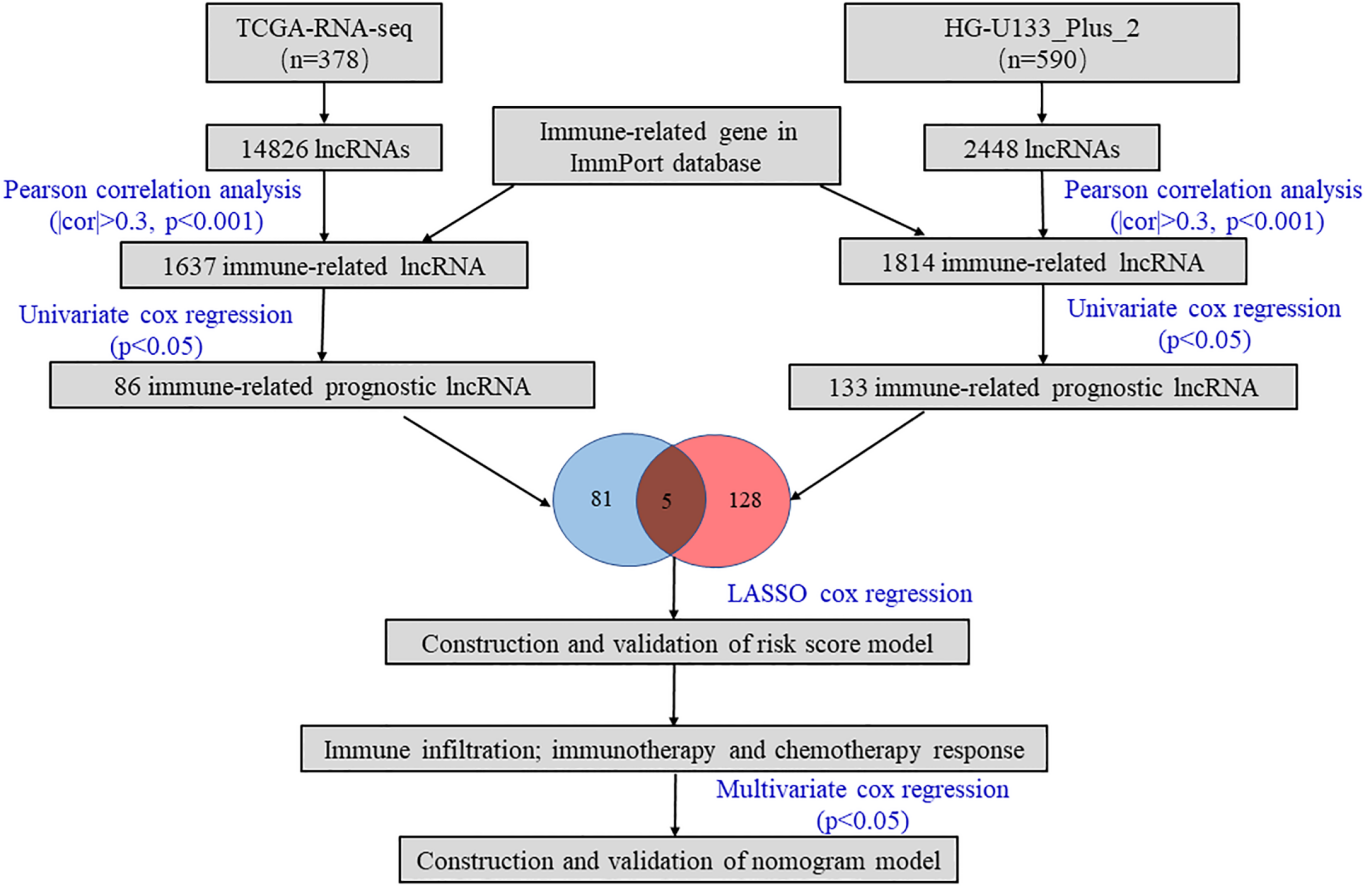
The workflow of this study.

**Figure 2:**
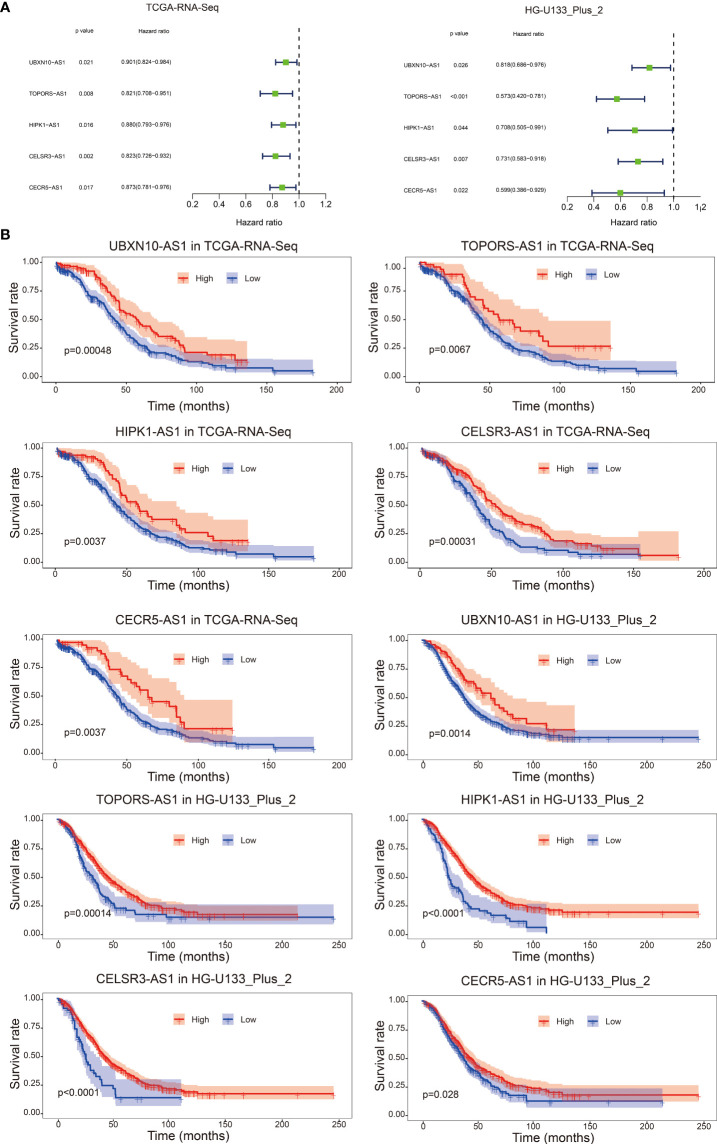
Forest plot of the prognostic ability of the five immune-related lncRNAs in TCGA-RNA-Seq cohort and HG-U133_Plus_2 cohort **(A)**; Kaplan–Meier curves suggested that expression of the five immune-related lncRNAs were associated with the OS in both TCGA-RNA-Seq cohort and HG-U133_Plus_2 cohort **(B)**.

As shown in **Figure 1**, we firstly identified 14826 lncRNAs in the TCGA-RNA-seq dataset and 2448 lncRNAs in the HG-U133_Plus_2 dataset, based on the lncRNA annotation file from GENCODE website. Then, the immune-related genes were download from the ImmPort database. Pearson correlation analysis was performed to screen the immune-related lncRNAs. The immune-related lncRNAs were identified as that the expression of lncRNAs were correlated with one or more of the immune-related genes (| cor | > 0.3 and *p < 0.001*). Finally, we obtained 1637 immune-related lncRNAs in TCGA-RNA-seq dataset and 1814 immune-related lncRNAs in the HG-U133_Plus_2 dataset, respectively (**Supplement Table 2**). To screen immune-related prognostic lncRNAs, the univariate Cox regression was implemented. The forest plot showed that 5 lncRNAs (UBXN10-AS1, TOPORS-AS1, HIPK1-AS1, CELSR3-AS1 and CECR5-AS1) were significantly correlated to prognosis of patients with ovarian cancer. All the lncRNAs were protective factors with hazard ratio (HR) <1 in both datasets (**Figure 2A**). The Kaplan–Meier curves confirmed that higher expression of all the five lncRNAs were associated with better OS in both cohorts (**Figure 2B**).

### Construction and validation of the immune-related lncRNA signature

In addition, we defined the TCGA-RNA-Seq dataset as discovery cohort and constructed an immune-related lncRNA signature. The risk score for each patient was calculated based on the coefficient for each lncRNA (**Supplement Figure 1**).

**Figure 3:**
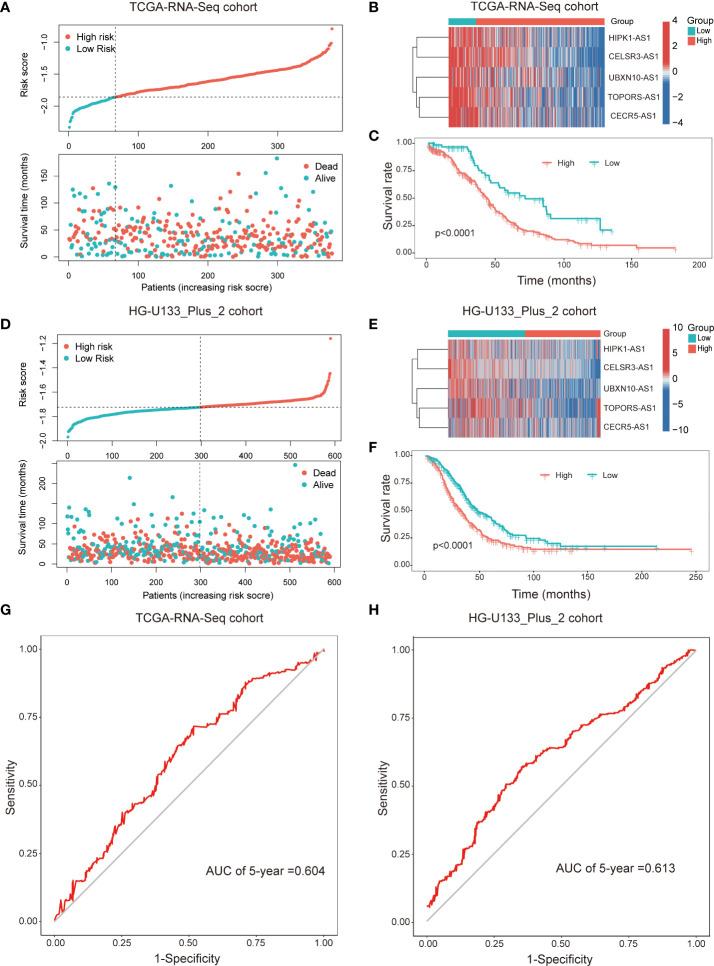
The immune-related prognostic signature was established and validated in TCGA-RNA-Seq cohort and HG-U133_Plus_2 cohort, respectively. Distributions of risk scores and survival status of OC patients in the TCGA-RNA-Seq cohort **(A)** and HG-U133_Plus_2 cohort **(D)**; Heat map analysis showed the association between risk score and the expression of the five lncRNAs in TCGA-RNA-Seq cohort **(B)** and HG-U133_Plus_2 cohort **(E)**; Kaplan–Meier curves showed that the high-risk subgroup had worse OS than the low-risk subgroup in TCGA-RNA-Seq cohort **(C)** and HG-U133_Plus_2 cohort **(F)**. ROC curves of the immune-related lncRNAs for predicting 5-year survival in TCGA-RNA-Seq cohort **(G)** and HG-U133_Plus_2 cohort **(H)**.

Subsequently, patients were divided into two subgroups dependent on the best cut-off value of risk score. The distributions of the risk score and survival status were listed in the **Figure 3A**. The heatmap showed that the expression of all the lncRNAs were higher in the low-risk group than in the high-risk group (**Figure 3B**). Kaplan-Meier survival curves indicated that patients with higher risk score had worse survival rate (*p<0.001*, **Figure 3C**). Furthermore, we validated the prognostic value of the immune-related lncRNA signature in the HG-U133_Plus_2 cohort. The results were consistent with the findings in the TCGA-RNA-Seq cohort. It’s suggested that the higher risk score was associated with shorter OS time and worse survival status (**Figures 3D-F**). The ROC curves demonstrated that the immune-related prognostic lncNRA signature harbored a promising ability to predict 5-year OS in the TCGA-RNA-Seq cohort and HG-U133_Plus_2 cohort (**Figures 3G, H**). All these demonstrated that the immune-related prognostic lncRNA signature might stably predict the survival outcome of patients with OC.

### Association between the prognostic signature and clinicopathological feathers

**Figure 4:**
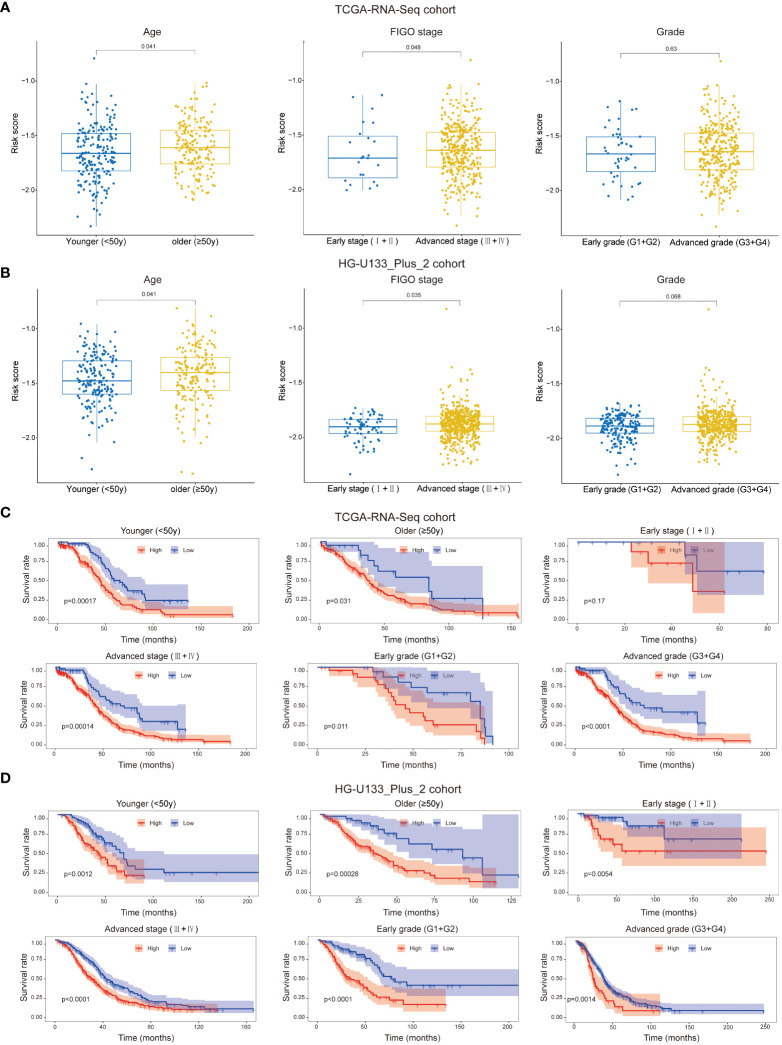
Patients with different clinicopathological features (including age, FIGO stage and Grade) had different levels of risk scores in TCGA-RNA-Seq cohort **(A)** and HG-U133_Plus_2 cohort **(B)**. Stratification analysis suggested that the immune-related lncRNAs signature retained its prognostic value in multiple subgroups in TCGA-RNA-Seq cohort **(C)** and HG-U133_Plus_2 cohort **(D)**. The younger and older group were divided based on 50y; FIGO I+II were identified as early stage and FIGO III+IV were identified as advanced stage; G1+G2 were identified as early grade and G3+G4 were identified as advanced grade.

We attempted to analyze the association between risk score and the clinicopathological feathers. It was suggested that patients with higher age and advanced FIGO stage had higher risk score, while the risk score was not associated with grade in both cohort (**Figures 4A, B**). Besides, we assessed the prognostic ability of the immune-related prognostic signature by performing a stratification analysis. Compared to patients with lower risk, patients with higher risk had worse OS in younger (<50y), older (≥50y), advanced FIGO stage (III+IV), early grade (G1+G2) and advanced grade (G3+G4) subgroups in the TCGA-RNA-Seq cohort (**Figure 4C**). Likewise, these results were validated in the HG-U133_Plus_2 cohort (**Figure 4D**).

Due to the small samples of the early FIGO stage (I+II) subgroup in TCGA-RNA-Seq cohort, there was no significant difference in OS between higher risk patients and lower risk patients (*p=0.17*, **Figure 4C**). However, we confirmed that the signature retained the ability to predict OS for patients with early stage in HG-U133_Plus_2 cohort (*p=0.0054*, **Figure 4D**). All these results revealed that it could be served as a potential predictor for OC patients.

### Modeling the prognostic nomogram

**Figure 5:**
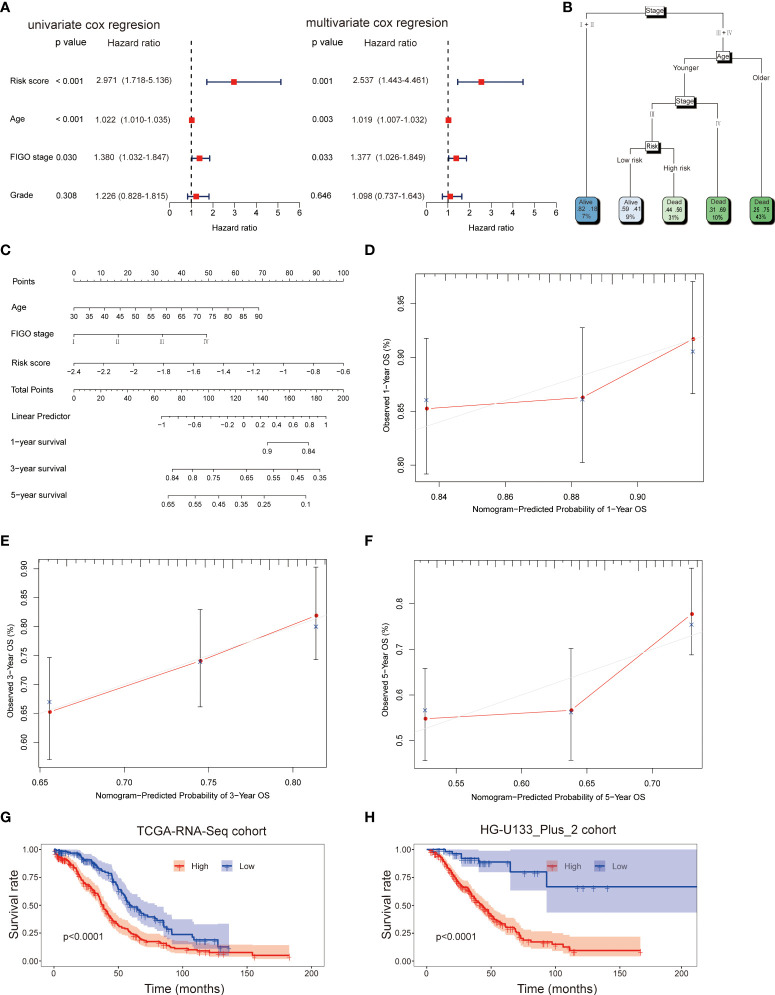
Univariate and multivariate analyses revealed that risk score was an independent prognostic predictor in the TCGA-RNA-Seq cohort **(A)**. Construction of decision tree based on risk score, age and stage. The younger and older subgroup were divided based on the median value of age **(B)**. Construction of nomogram based on risk score, age and stage **(C)**. Calibration plots of the nomogram for predicting the probability of OS at 1, 3, and 5-years in the TCGA-RNA-Seq cohort **(D–F)**; KM survival plot analysis showed that patients with high-risk had a worse OS than patients with low-risk subgroup in both TCGA-RNA-Seq cohort and HG-U133_Plus_2 cohort **(G, H)**.

Firstly, the independent prognostic factors were identified by using the univariate and multivariant cox regression in the TCGA-RNA-Seq cohort. The univariate cox regression analysis indicated that risk score (HR: 2.971; 95% CI: 1.718-5.136; *p<0.001*), age (HR: 1.022; 95% CI: 1.010-1.035; *p<0.001*), stage (HR: 1.380; 95% CI: 1.032-1.847; *p=0.030*) but not grade (HR: 1.226; 95% CI: 0.828-1.815; *p=0.308*) were associated with OS of patients (**Figure 5A**). Multivariate cox analysis further proved that risk score (HR: 2.537; 95% CI: 1.443-4.461; *p=0.001*), age (HR: 1.019; 95% CI: 1.007-1.032; *p=0.003*) and stage (HR: 1.377; 95% CI: 1.026-1.849; *p=0.033*) were independent prognostic factors for OC patients (**Figure 5A**). Therefore, age, FIGO stage and risk score were applied to build a decision tree with five different risk subgroups (**Figure 5B**). The split at the top of the tree resulted in two large branches: the left-hand branch included patients with early stage; the right-hand branch corresponded to patients with advanced stage. The right branch is further subdivided by age, stage and risk score. Overall, the tree had five terminal nodes, leading to partitioning OC patients in five subgroups. It worth mentioning that compared to patients with younger age (<50y), stage III and high-risk score (31% of overall samples), patients with younger age (<50y), stage III and low-risk score (9% of overall samples) showed higher alive probability (44% *vs* 59%). In order to make the signature more applicable in clinic, a nomogram based on the predictors (including risk score, age and FIGO stage) was established in the TCGA-RNA-Seq cohort (**Figure 5C**). Calibration plots showed that the observed *vs* predicted rates of 1-, 3- and 5-year OS showed perfect concordance (**Figures 5D-F**). Moreover, the predictive performance of the nomogram was evaluated by the ROC curve. Compared to other predictors (including age and FIGO stage), the model’s 5-year AUC values were higher in both TCGA-RNA-Seq cohort and HG-U133_Plus_2 cohort (**Supplement Figure 2**). KM survival plot analysis showed that patients with high-risk had a worse OS than patients with low-risk subgroup in both TCGA-RNA-Seq cohort and HG-U133_Plus_2 cohort (*p<0.001*, *p<0.001*, respectively, **Figures 5G, H**). These data confirmed that the nomogram had a robust and stable ability to predict the OS for OC patients.

### Association between the prognostic signature and immune infiltration and immunotherapy response

**Figure 6:**
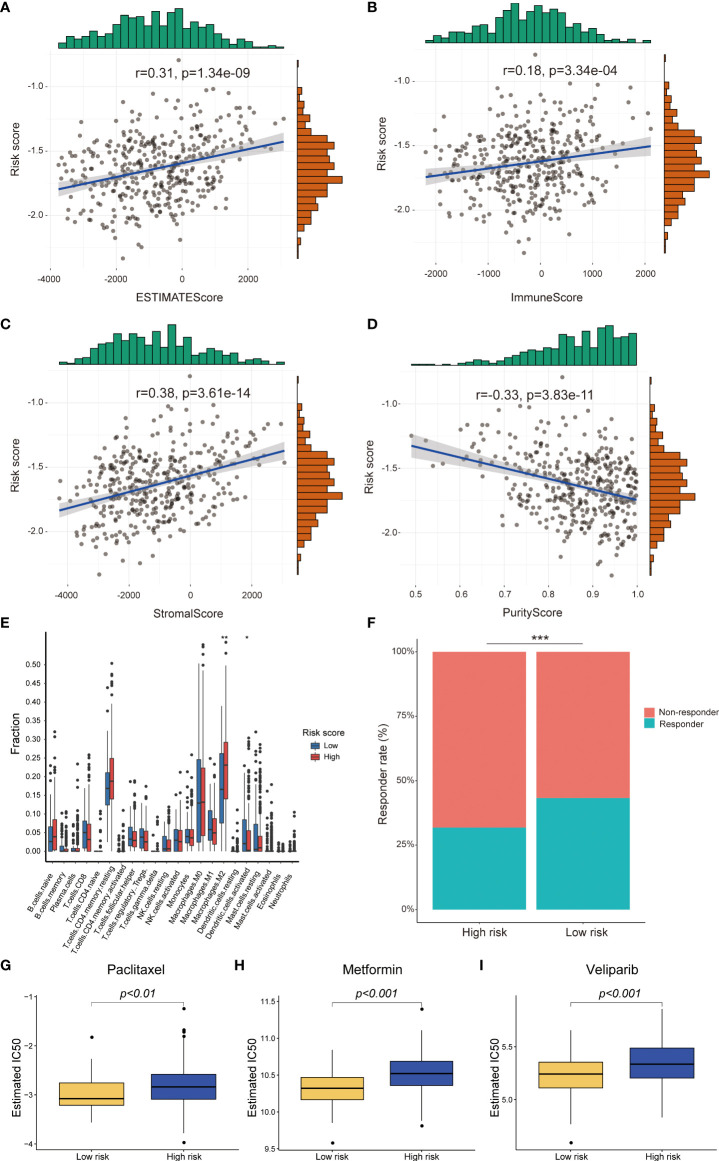
Difference between high-risk score group and low-risk score group in immune infiltration, immunotherapy and chemotherapy response prediction in TCGA-RNA-Seq cohort. The risk score was positively correlated with EstimateScore, ImmuneScore, StromalScore and negatively correlated with PurityScore **(A-D)**; The different infiltrated fraction of 22 immune cells between high-risk group and low-risk group **(E)**; The immunotherapy response of patients with OC in high- and low-risk subgroups **(F)**; Estimated IC50 values indicated the chemotherapy response of paclitaxel, metformin and veliparib in TCGA-RNA-Seq cohort **(G-I)**. *p < 0.05; **p < 0.01; ***p < 0.001.

To explore the influence of risk score on immune infiltration and immunotherapy response, the ESTIMATEscore, ImmuneScore, PurityScore and StromalScore were calculated to explain immune cell and stromal cell infiltration situation. The correlation analysis results indicated that the risk score was positively correlated with the ESTIMATEscore, ImmuneScore and StromalScore, but negatively correlated PurityScore in TCGA-RNA-Seq cohort (**Figures 6A-D**). The similar results were validated in the HG-U133_Plus_2 cohort (**Supplement Figures 3A-D**). After that, the distribution proportion of 22 immune cells in high-risk group and low-risk group were analyzed. In TCGA-RNA-Seq cohort, the distribution proportion of Macrophages cells was higher in high-risk group than low-risk group, whereas the distribution proportion of activated dendritic cells were significantly lower (**Figure 6E**). In the HG-U133_Plus_2 cohort, not only Macrophages cells and activated dendritic cells but also memory B cells, plasma cells, CD4^+^ T cells, Treg cells, NK cells, activated mast cells and neutrophils were differently distributed in high-risk group and low-risk group. (**Supplement Figure 3E**). Besides, the potential response to immunotherapy for each patient was assessed by using the TIDE algorithm. The results suggested that patients with low-risk score were more sensitive to immunotherapy than those with high-risk score in TCGA-RNA-Seq cohort (*p<0.001*, **Figure 6F**). Taken together, these results indicated that patients with high-risk score might recruit multiple immune cells and stromal cells and facilitate OC immune surveillance evasive.

### Analysis the correlation between the risk model and chemotherapy response

Until now, chemotherapy is the main treatment method for OC patients. Therefore, we tried to identify the association between the risk score and chemotherapy response in both TCGA-RNA-Seq cohort and HG-U133_Plus_2 cohort (**Supplement Table 3**). We revealed that a higher risk score was associated with a lower IC50 of chemotherapeutics such as paclitaxel (*p<0.01*), metformin (*p<0.001*) and veliparib (*p<0.001*) in TCGA-RNA-Seq cohort (**Figures 6G-I**). In HG-U133_Plus_2 cohort, the risk score was also confirmed to be negatively associated with IC50 of paclitaxel (*p<0.05*), metformin (*p<0.001*), and veliparib (*p<0.05*), whereas it was positively associated with the IC50 of cisplatin (**Supplement Figures 3F-I**), which indicated that the model acted as a potential predictor for chemosensitivity.

### Exploration of the five immune-related lncRNA function

**Figure 7:**
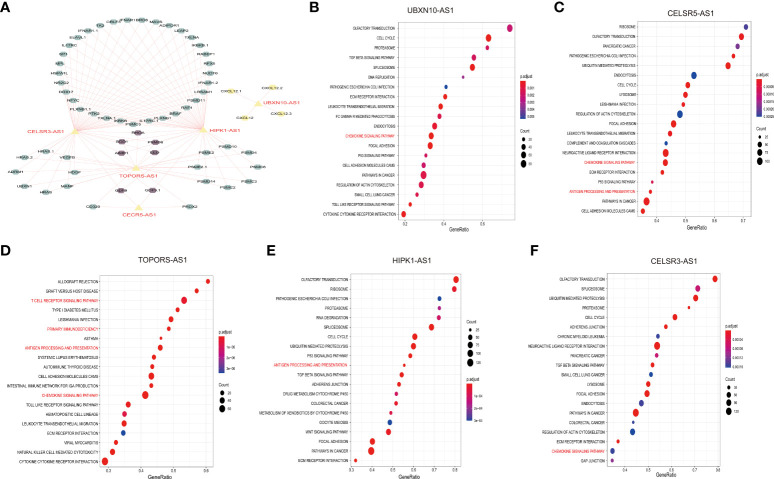
Exploration of the five immune-related lncNRAs function. Construction of the co-expression network of the five immune-related lncRNAs and immune-related mRNA **(A)**. GSEA assay to explore the pathways associated with the five immune-related lncRNAs **(B-F)**.

To further understand the function of the five immune-related lncRNA, we constructed the co-expression network between the five immune-related lncRNA and immune-related mRNA. As shown in **Figure 7A**, CELSR3-AS1 and HIPK1-AS1 showed most connections with immune-related mRNAs. Besides, GSEA analysis was performed to further explore and interpret the enrichment results. The annotated top20 pathways were listed in **Figures 7B-F**. As shown in the bubble charts, all the five lncRNAs, especially TOPORS-AS1, were significantly associated with immune-related pathways. UBXN10-AS1, TOPORS-AS1, CELSR3-AS1 and CECR5-AS1 were significantly associated with chemokine signaling pathway. Except that, TOPORS-AS1, CECR5-AS1 and HIPK1-AS1 participate in antigen processing and presentation. In addition, the associations between lncRNA expression and individual immune cell subtypes were computed by Spearman correlation in TCGA-RNA-Seq cohort and HG-U133_ Plus_2 cohort (**Supplement Figures 4A, B**). Moreover, there is a significant difference in actin binding, adaptive of immune response based on somatic recombination of immune receptors built from immunoglobulin superfamily domains, antigen receptor mediated signaling pathway and B cell activation between high-risk group and low-risk group in both TCGA-RNA- seq cohort and the HG-U133_Plus_2 cohort (**Supplement Figures 4C, D**). All these results indicated that the five lncRNAs might affect immune infiltration and facilitate ovarian cancer immune surveillance evasive by regulating immune-related pathways in OC.

### Overexpression of UBXN10-AS1 suppressed cell proliferation and migration in OC cell lines

**Figure 8:**
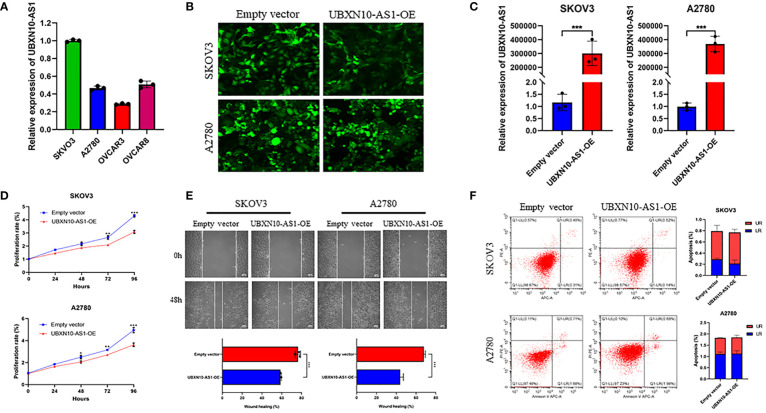
UBXN10-AS1 significantly suppressed cell proliferation and inhibited cell migration in SKOV3 and A2780 cell lines. Expression of UBXN10-AS1 in SKOV3, A2780, OVCAR3 and OVCAR8 cell lines **(A)**; Overexpression of UBXN10-AS1 in SKOV3 and A2780 cell lines **(B, C)**; Overexpression of UBXN10-AS1 significantly suppressed cell proliferation in SKOV3 and A2780 cell lines **(D)**; Overexpression of UBXN10-AS1 significantly inhibited cell migration in SKOV3 and A2780 cell lines **(E)**; Overexpression of UBXN10-AS1 had no influence on apoptosis in SKOV3 and A2780 cell lines **(F)**. *p<0.05; **p<0.01; ***p<0.001.

To figure out the function of LncRNAs in OC, the expression of lncRNAs in OC cell lines were detected. Due to the low abundance of CELSR3-AS1, CECR5-AS1 and HIPK1-AS1, they were not detected in A2780, SKOV3, OVCAR8 and OVCAR8 cell lines. UBXN10-AS1 were more highly expressed in SKOV3 and A2780 cell lines (**Figure 8A**). Thus, the function of UBXN10-AS1, as the candidate gene, were further studies in A2780 and SKOV3 cell lines (**Figures  8B, C**). CCK8 assay revealed that overexpression of UBXN10-AS1 significantly suppressed cell proliferation (**Figure 8D**). Besides, it could also inhibit the cell migration of SKOV3 and A2780 (**Figure 8E**). However, UBXN10-AS1 overexpression had no influence on cell apoptosis (**Figure 8F**). All these results indicated that UBXN10-AS1 might serve as a tumor suppressor in OC.

## Discussion

Due to the heterogeneity of OC, it is difficult to blame it on a single specific issue (56). Recently, gene signatures developed by the combination of high-throughput sequencing technology and bioinformatics have been widely used in individualized therapy and prognosis evaluation, which have the better prediction ability than a single biomarker (57). Multiple evidence demonstrated that immune systems made an important contribution to cancer initiation, development, metastasis, and immune escape (58–60). Furthermore, more and more immune-related lncRNAs signatures had been successfully developed and had a perfect prediction accuracy for survival and prognosis in various tumors (61, 62). However, the prediction value of immune-related lncNRAs signature in OC has not been explored.

In our study, we firstly screened immune-related lncRNAs in OC patients from the TCGA-RNA- seq dataset (n = 378) and the HG-U133_Plus_2 dataset (n = 590) by using Pearson correlation analysis. Afterwards, the prognostic significance of immune-related lncRNAs were identified by using univariate cox regression analysis. Finally, five immune-related lncRNAs (including UBXN10-AS1, TOPORS-AS1, HIPK1-AS1, CELSR3-AS1 and CECR5-AS1) were demonstrated to serve as prognostic biomarkers in both TCGA-RNA-seq dataset and the HG-U133_Plus_2 dataset. Recently, it was reported that overexpression of TOPORS-AS1 suppressed cell proliferation and inhibited aggressive cell behaviors, including migration, invasion, and colony formation *via* inhibiting the Wnt/β-catenin pathway in ovarian cancer cells. Moreover, OC patients with high TOPORS-AS1 expression had favorable OS compared to low expression, which was consistent with our study (63). In gastric cancer, it was also proved that the expression of TOPORS-AS1 and its associated gene, NDUFB6 in gastric cancer tissues were significantly lower than that in adjacent tissues (64). All the evidence indicated that TOPORS-AS1 might play important roles in carcinogenesis. Unfortunately, the function of UBXN10-AS1, HIPK1-AS1, CELSR3-AS1 and CECR5-AS1 in OC have not been reported. In colon adenocarcinoma, UBXN10-AS1 was expressed with low level and overexpression of UBXN10-AS1 suppressed tumor growth *in vivo* and *in vitro (* 65). The function of UBXN10-AS1 in OC has not been reported. Therefore, we explored the function of UBXN10-AS1 in cell proliferation and migration in SKOV3 and A2780 cell lines. The results indicated that UBXN10-AS1 could significantly reduce cell proliferation and migration in OC.

Furthermore, we constructed an immune-related lncRNA prognostic signature to predict the OS. Based on the best cutoff value of risk score, all patients were divided into high- and low-risk groups. There was significantly different in OS between both high-risk group and low-risk group. Stratified analysis results revealed that the risk score was associated with age and FIGO stage. By using multivariate cox regression, we demonstrated that risk score was an independent prognostic factor for OC patients. In order to make the signature more applicable in clinic, a nomogram was established. Besides, the potential role of the immune-related signature in immune infiltration and immunotherapy response were investigated. The results indicated that various immune cells, especially tumor associated macrophages (TAMs), were differently distributed in high-risk group and low-risk group. Previous study reported that M2-like TAMs accelerated tumor growth, promoted tumor cell invasion and metastasis, and inhibited immune killing to promote tumor progression, which was consistent with our study (66). Accumulating evidence demonstrated that immune systems make a crucial contribution to the antitumor effects of conventional chemotherapy-based and radiotherapy-based cancer treatments (67, 68). Furthermore, the association between risk model and chemotherapy response were investigated. Our results suggested that the risk model might serve as potential predictor for chemosensitivity of various antitumor drugs, especially for paclitaxel, metformin, and veliparib, which are commonly used in treating OC patients.

In our study, both TCGA-RNA- seq datasets and HG-U133_Plus_2 datasets were included. The sample size is much larger than the studies before, which makes it more robust and reliable. However, there are some limitations. Due to different platforms, gene expression values are subject to sampling bias. Additionally, the roles of the lncRNAs and their interactions with immune-related genes are not confirmed using *in vitro* and *in vivo* experiments.

In summary, we have constructed a novel immune-related lncRNA signature, which have a potential prognostic value for ovarian cancer patients and might facilitate personalized counselling for immunotherapy and chemotherapy. Prospective studies are needed to further validate its predictive accuracy for estimating prognoses of ovarian cancer.

## Data availability statement

The datasets presented in this study can be found in online repositories. The names of the repository/repositories and accession number(s) can be found in the article/**Supplementary Material**.

## Author contributions

HL wrote the manuscript. Z-YL contributed to the data collection and analysis. NW and JW designed the idea and design the study. All authors contributed to the article and approved the submitted version.

## Funding

This research was supported by the National Natural Science Foundation of China (NO.81972836), National Key R&D Program (2016YFC1303703) and the Science and Technology Innovation Program of Hunan Province (2020RC2065), the Youth Natural Science Foundation of Hunan Province (2021JJ40321).

## Acknowledgments

We would like to thank Dr. Quan Cheng for the bioinformatics technology support.

## Conflict of interest

The authors declare that the research was conducted in the absence of any commercial or financial relationships that could be construed as a potential conflict of interest.

## Publisher’s note

All claims expressed in this article are solely those of the authors and do not necessarily represent those of their affiliated organizations, or those of the publisher, the editors and the reviewers. Any product that may be evaluated in this article, or claim that may be made by its manufacturer, is not guaranteed or endorsed by the publisher.

## Supplementary material

Supplementary Figure 1 Least absolute shrinkage and selection operator (LASSO) regression was performed, calculating the minimum criteria **(A, B)** and coefficients **(C).**

Supplementary Figure 2 Time-dependent receiver operating characteristic (ROC) curves to predict the 5-year OS for the risk score, age and stage in TCGA-RNA-Seq cohort **(A)** and HG-U133_Plus_2 cohort **(B)**.

Supplementary Figure 3 Difference between high-risk score group and low-risk score group in immune infiltration, immunotherapy and chemotherapy response prediction in HG-U133_Plus_2 cohort. The risk score was positively correlated with EstimateScore, ImmuneScore, StromalScore and negatively correlated with PurityScore **(A-D)**; The Association of the signature and the distribution of 22 immune cells **(E)**; Estimated IC50 values indicated the chemotherapy response of paclitaxel, metformin, veliparib and Cisplatin in TCGA-RNA-Seq cohort **(F–I)**. **p < 0.05*; ***p < 0.01*; ****p < 0.001*.

Supplementary Figure 4 Molecular immune cell subtypes related to the five immune-related lncRNAs in HG-U133_Plus_2 cohort **(A)** and TCGA-RNA-Seq cohort **(B)**, respectively. GSEA identify the different pathways between high-risk group and low-risk group in HG-U133_Plus_2 cohort **(C)** and TCGA-RNA-Seq cohort **(D)**, respectively.

Supplementary Table 1 The primers in the study.

Supplementary Table 2 The immune-related lncRNAs identified in TCGA-RNA-seq dataset and HG-U133_Plus_2 dataset.

Supplementary Table 3 The chemotherapy response to anticancer drugs commonly used to treating cancers in TCGA-RNA-seq dataset and HG-U133_Plus_2 dataset.
