# Supplementary figures and images for: Identification and validation of an immune-related lncRNAs signature to predict the overall survival of ovarian cancer
Source: Front Oncol. 2022 Oct 12;12:999654. doi: 10.3389/fonc.2022.999654 (PMC9596922; doi:10.3389/fonc.2022.999654)

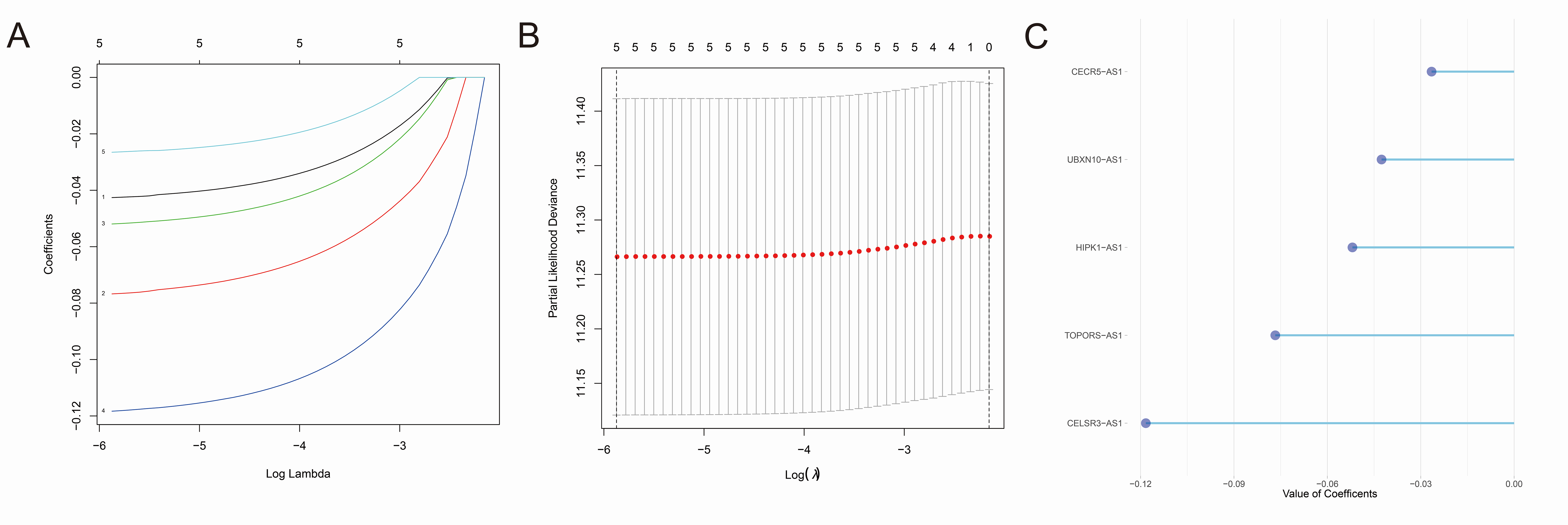

Supplement: Supplementary Figure 1 — Least absolute shrinkage and selection operator (LASSO) regression was performed, calculating the minimum criteria (A, B) and coefficients (C). [file Image_1.jpeg]

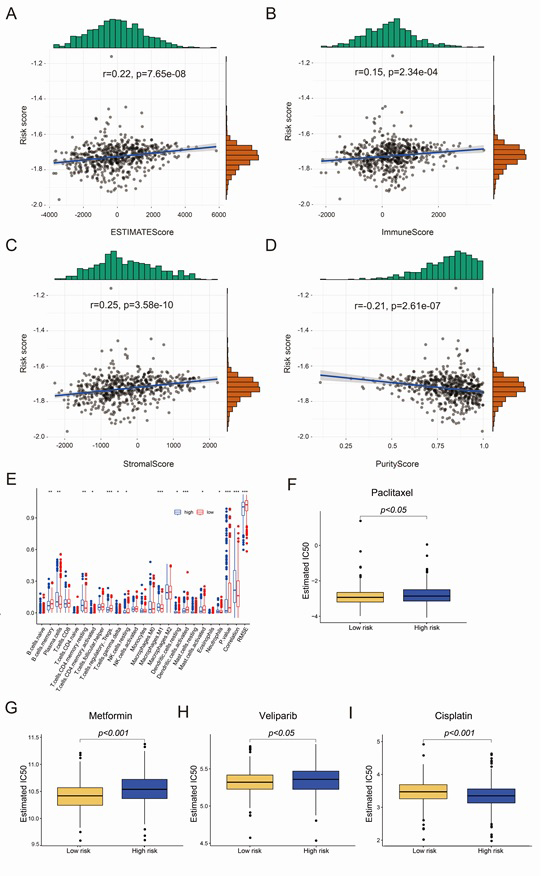

Supplement: Supplementary Figure 3 — Difference between high-risk score group and low-risk score group in immune infiltration, immunotherapy and chemotherapy response prediction in HG-U133_Plus_2 cohort. The risk score was positively correlated with EstimateScore, ImmuneScore, StromalScore and negatively correlated with PurityScore (A-D); The Association of the signature and the distribution of 22 immune cells (E); Estimated IC50 values indicated the chemotherapy response of paclitaxel, metformin, veliparib and Cisplatin in TCGA-RNA-Seq cohort (F–I). *p < 0.05; **p < 0.01; ***p < 0.001. [file Image_3.jpeg]
