# Supplementary material for: Identification and validation of an immune-related lncRNAs signature to predict the overall survival of ovarian cancer
Source: Front Oncol. 2022 Oct 12;12:999654. doi: 10.3389/fonc.2022.999654 (PMC9596922; doi:10.3389/fonc.2022.999654)
Supplement: Supplementary Table 1 — The primers in the study. [file DataSheet_1.docx]

Supplement Table 1. The primers used in the study.

| Genes | Sequences |
| --- | --- |
| GAPDH | Forward primer: 5ʹ-GGAGCGAGATCCCTCCAAAAT-3ʹ |
|  | Reverse primer: 5ʹ- GGCTGTTGTCATACTTCTCATGG-3ʹ |
| UBXN10-AS1 | Forward primer: 5ʹ-GTTGCATAGGTCCCTCGGTT-3ʹ |
|  | Reverse primer: 5ʹ-AGACGAGCAGAAACACCACC-3ʹ |
| TOPORS-AS1 | Forward primer: 5ʹ- CTGGGAGGTTACTGTAAGGC -3ʹ |
|  | Reverse primer: 5ʹ- GCAAGCAGCAACACTGAATAA -3ʹ |
| HIPK1-AS1 | Forward primer: 5ʹ- GTTCCTGAAGTGGGGGACAT-3ʹ |
|  | Forward primer: 5ʹ- TTTCGATGGTGACGGACAGT-3ʹ |
| CELSR3-AS1 | Forward primer: 5ʹ- CCAATCGCTCCCAACTAGAGG-3ʹ |
|  | Forward primer: 5ʹ- TCTCCTGGTCTTCTGATCGC-3ʹ |
| CECR5-AS1 | Forward primer: 5ʹ- GCGCGCCAATTGTTAAGTCT-3ʹ |
|  | Forward primer: 5ʹ- GGTGTCCTTGGCACAGGTAT-3ʹ |
